# A follow‐up questionnaire survey 2022 on radiation protection among 464 medical staff from 34 endoscopy–fluoroscopy departments in Japan

**DOI:** 10.1002/deo2.227

**Published:** 2023-04-13

**Authors:** Shiro Hayashi, Mamoru Takenaka, Hirofumi Kogure, Takayuki Yakushijin, Yousuke Nakai, Kenji Ikezawa, Shinjiro Yamaguchi, Toshio Fujisawa, Yuzuru Tamaru, Iruru Maetani, Hirotsugu Maruyama, Satoshi Asai, Tadayuki Takagi, Koji Nagaike, Yasuki Hori, Tetsuya Sumiyoshi, Hidetaka Tsumura, Hisashi Doyama, Toshiyuki Yoshio, Kazuo Hara, Seiichiro Abe, Ichiro Oda, Motohiko Kato, Hiroko Nebiki, Tatsuya Mikami, Masanori Miyazaki, Kazuhiro Matsunaga, Makoto Hosono, Tsutomu Nishida, REX‐GI Study Group, Satoshi Egawa, Akihiro Nishihara, Ken Ohnita, Ryuki Minami, Naoya Tada, Katsumasa Kobayashi, Masayuki Kato

**Affiliations:** ^1^ Department of Gastroenterology and Internal Medicine Hayashi Clinic Osaka Japan; ^2^ Department of Gastroenterology Toyonaka Municipal Hospital Osaka Japan; ^3^ Department of Gastroenterology and Hepatology Kindai University Faculty of Medicine Osaka Japan; ^4^ Division of Gastroenterology and Hepatology Department of Medicine Nihon University School of Medicine Tokyo Japan; ^5^ Department of Gastroenterology and Hepatology Osaka General Medical Center Osaka Japan; ^6^ Department of Gastroenterology Graduate School of Medicine The University of Tokyo Tokyo Japan; ^7^ Department of Endoscopy and Endoscopic Surgery The University of Tokyo Hospital Tokyo Japan; ^8^ Department of Hepatobiliary and Pancreatic Oncology Osaka International Cancer Institute Osaka Japan; ^9^ Department of Gastroenterology and Hepatology Kansai Rosai Hospital Hyogo Japan; ^10^ Department of Gastroenterology Juntendo University, Hongo Campus Tokyo Japan; ^11^ Department of Gastroenterology National Hospital Organization Kure Medical Center and Chugoku Cancer Center Hiroshima Japan; ^12^ Division of Gastroenterology and Hepatology Department of Internal Medicine Toho University Ohashi Medical Center Tokyo Japan; ^13^ Department of Gastroenterology Osaka Metropolitan University Osaka Japan; ^14^ Department of Gastroenterology Tane General Hospital Osaka Japan; ^15^ Department of Gastroenterology Fukushima Medical University School of Medicine Fukushima Japan; ^16^ Department of Gastroenterology and Hepatology Suita Municipal Hospital Osaka Japan; ^17^ Department of Gastroenterology and Metabolism Nagoya City University Graduate School of Medical Sciences Aichi Japan; ^18^ Department of Gastroenterology Tonan Hospital Hokkaido Japan; ^19^ Department of Gastroenterological Oncology Hyogo Cancer Center Hyogo Japan; ^20^ Department of Gastroenterology Ishikawa Prefectural Central Hospital Ishikawa Japan; ^21^ Department of Gastroenterology Cancer Institute Hospital Tokyo Japan; ^22^ Department of Gastroenterology Aichi Cancer Center Aichi Japan; ^23^ Endoscopy Division National Cancer Center Hospital Tokyo Japan; ^24^ Department of Internal Medicine Kawasaki Rinko General Hospital Kanagawa Japan; ^25^ Department of Gastroenterology Keio University School of Medicine Graduate School of Medicine Tokyo Japan; ^26^ Department of Gastroenterology Osaka City General Hospital Osaka Japan; ^27^ Department of Gastroenterology Hirosaki University Aomori Japan; ^28^ Department of Gastroenterology and Hepatology Osaka Police Hospital Osaka Japan; ^29^ Department of Gastroenterological Endoscopy Kanazawa Medical University Ishikawa Japan; ^30^ Department of Radiology Kindai University Faculty of Medicine Osaka Japan

**Keywords:** endoscopy staff, fluoroscopy, Japan, questionnaire, radiation protection

## Abstract

**Objectives:**

We surveyed and reported low protective equipment usage and insufficient knowledge among endoscopy‐fluoroscopy departments in Japan in 2020. Two years later, we conducted a follow‐up survey of doctors, nurses, and technologists in Japan.

**Methods:**

We conducted a questionnaire survey on radiation protection from May to June 2022. The participants were medical staff, including doctors, nurses, and radiological and endoscopy technicians in endoscopy‐fluoroscopy departments. The questionnaire included 17 multiple‐choice questions divided into three parts: background, equipment, and knowledge.

**Results:**

We surveyed 464 subjects from 34 institutions. There were 267 doctors (58%), 153 nurses (33%), and 44 technologists (9%). The rate of wearing a lead apron was 98% in this study. The rates of wearing a thyroid collar, lead glasses, and radiation dosimeter were 27%, 35%, and 74%, respectively. The trend of the protective equipment rate was similar to that of our previous study; however, radiation dosimetry among doctors was still low at 58%. The percentage of subjects who knew the radiation exposure (REX) dose of each procedure was low at 18%. Seventy‐six percent of the subjects attended lectures on radiation protection, and 73% knew about the three principles of radiation protection; however, the concept of diagnostic reference levels was not well known (18%). Approximately 60% of the subjects knew about the exposure dose increasing cancer mortality (63%) and the 5‐year lens REX limit (56%).

**Conclusions:**

There was some improvement in radiation protection equipment or education, but relatively little compared to the 2020 survey of endoscopy departments.

## INTRODUCTION

The International Commission on Radiological Protection (ICRP) has stated that medical staff involved in radiation‐related procedures should have appropriate knowledge of radiation protection.^1^, ^2^ Gastroenterological societies have issued similar guidelines, but not in Japan, where awareness and protection rates remain low in clinical practice.^3^, ^4^, ^5^, ^6^, ^7^ In response to the rapid increase in cardiovascular interventional procedures and computed tomography examinations, the Japanese Society of Cardiology (JCS) published the “JCS Guidelines for radiation exposure (REX) in cardiovascular diseases” in 2006. The guidelines were revised in 2011, and the third edition was published in 2021.^8^

In Japan, on March 11, 2019, the Ordinance for Enforcement of the Medical Care Act was amended because of the importance of medical radiation control. The ordinance, regarding the development of a safety management system for medical radiation, became effective on April 1, 2020. It requires all hospitals and clinics equipped with X‐ray equipment to appoint a medical radiation safety manager, establish guidelines for the safety management of medical radiation, provide safety training for staff, share information with those who receive radiation treatment, and, depending on the equipment in their possession, establish a system for the management and control of medical radiation doses and REX.

With this background, we conducted a survey on radiation protection for medical staff in endoscopy departments in Japan in 2020. This study revealed that staff did not have enough radiation protection equipment or education.^9^ We speculate that this may be due to insufficient awareness‐raising activities by gastroenterology‐related societies in Japan. We reported in 2022 that the REX from gastrointestinal fluoroscopic procedures (REX‐GI) study aimed to establish diagnostic reference levels (DRLs) for the following interventional procedures in GI endoscopy units from 23 hospitals all around Japan.^10^, ^11^ Although several surveys of physicians' or healthcare workers' attitudes toward medical exposure have been reported,^6^ few studies have examined changes over time. In the present study, we conducted a follow‐up survey and studied DRL penetration rates among doctors, nurses, and technologists from endoscopy‐fluoroscopy departments in Japan.

## MATERIALS AND METHODS

The study period was from May 2022 to June 2022. We e‐mailed participants in a previous study,^9^ the REX‐GI study^10^ and the Fight Japan study group, and enrolled doctors, nurses, and radiological and endoscopy technicians in each fluoroscopic endoscopy suite of the previous participating facilities. The participants provided informed consent at the beginning of the survey and answered an anonymous, online questionnaire using Google Forms.

The questionnaire used in the survey included many of the same questions as the previous survey^9^ and new questions. These questions were divided into the following three categories: background, equipment, and knowledge. The details of the questionnaire are shown in Table 1. Questions 1–6 addressed the background of each person or institution. Questions 7–10 asked about the proper equipment for radiation protection. Questions 11–17 focused on knowledge of REX and protection. Fourteen of 17 questions were the same as in the previous survey. We added three additional questions on awareness of DRLs, the estimated REX dose to increase cancer mortality, and the revised eye lens radiation dose limit.

**TABLE 1:** Questions and answers (participants’ responses were anonymous).

**Question**	**Answer**
1. What is your sex?	a) Female and b) male
2. How old are you?	a) Twenties, b) thirties, c) forties, d) fifties, and e) over sixty
3. What is your job title?	a) Medical doctor, b) nurse, and c) technologist
4. What is the size of your institution?	a) University hospital or medical center, b) Regional general hospital (>300 beds), and c) Other
5. How many years of career experience do you have?	a) 1–5, b) 6–10, c) 11–15, d) 16–20, and e) Over 21 years
6. Do you operate the fluoroscopy unit?	a) Yes and b) no
7. Do you always wear a lead apron?	a) Yes and b) no
8. Do you always wear a thyroid collar?	a) Yes and b) no
9. Do you always wear lead glasses?	a) Yes and b) no
10. Do you always wear a radiation dosimeter?	a) Yes and b) no
11. What type is your fluoroscopy unit, an undercouch or overcouch C‐arm system?	a) Undercouch (exposure from below), b) overcouch (expose from above), and c) I do not know
12. Do you know how much radiation dose you are exposed to in each endoscopic procedure under fluoroscopy?	a) Yes and b) no
13. Have you ever attended a basic lecture on radiation exposure?	a) Yes and b) no
14. Do you know the three principles of radiation protection?	a) Yes and b) no
15. Do you know about DRL (diagnostic reference level)?	a) Yes and b) no
16. Do you know about the radiation exposure dose which is estimated to increase cancer mortality by 0.5%?	a) 1, b) 10, c) 100, and d) 1000 mSv
17. Do you know about the revised lens exposure dose 5‐year limit?	a) 1, b) 10, c) 100, and d) 1000 mSv

### Statistical analysis

The categorical variables are expressed as the number in each category or the frequency and were compared using the chi‐square test or Fisher's exact test when appropriate. In statistical comparisons between the two groups, a *p*‐value of 0.05 was considered to indicate statistical significance. In statistical comparisons among the three groups, the Bonferroni method was used to study how the familywise error rate could be adjusted. We used the Cochran‐Armitage trend test to evaluate the trend of the proportion of age group and career experience. *p*‐Values were two‐sided, and a *p*‐value < 0.017 was considered statistically significant. All statistical analyses were performed with JMP software (ver. 16.2; SAS Institute, Inc., Cary, NC, USA).

## RESULTS

### Responses to the questionnaire

We e‐mailed survey invitations to 117 institutions. We obtained answers from 466 subjects, including endoscopists, nurses, and technicians, from 34 institutions (participating institution rate: 34/117, 29%). Two subjects accessed the internet survey but did not consent. Ultimately, a total of 464 subjects were included in this follow‐up survey.

Questions 1–6 regarding the background of each person or institution: There were 265 (57%) males. Most of the subjects were in their 30s (174, 38%) and 40s (136, 29%). Doctors were the most common occupation (267, 58%). Two hundred eighty‐one subjects worked at university hospitals or medical center hospitals (60%), 147 worked at regional general hospitals (>300 beds; 32%), and the other 36 worked at other types of hospitals (8%). Regarding years of experience, 82 (18%) had 1–5 years, 79 (17%) 6–10 years, 95 (20%) 11–15 years, 71 (15%) 16–20 years, and 137 (30%) more than 21 years. Three hundred and twenty‐five subjects (70%) had operated fluoroscopy units by themselves.

Questions 7–10 regarding wearing protective equipment: Four hundred fifty‐seven subjects (98%) always wore a lead apron, 123 subjects (27%) wore a thyroid collar, 164 subjects (35%) wore lead glasses, and 344 subjects (74%) wore a radiation dosimeter.

Questions 11–17 regarded education and knowledge of radiation protection: 54 subjects (12%) did not know about the radiation output system of fluoroscopy equipment. Eighty‐four subjects (18%) were aware of the radiation dose of each procedure, 351 subjects (76%) had received lectures on radiation protection, 338 subjects (73%) were aware of the three principles of radiation protection, 84 subjects (18%) knew about DRL, 292 subjects (63%) knew the REX dose estimated to increase cancer mortality by 0.5%, and 262 subjects (56%) knew the revised eye lens exposure dose limit. (Table 2)

**TABLE 2:** Answers from all subjects.

Questions	Answers	All *n* = 464	Doctors *n* = 267	Nurses *n* = 153	Technologists *n* = 44
1. Sex, *n* (%)	Male	265, 57%	221, 83%	12, 8%*	32, 73%
	Female	199, 43%	46, 17%	141, 92%*	12, 27%
2. Age group, *n* (%)	20–29	74, 16%	41, 15%	23, 15%	10, 23%
	30–39	174, 38%	123, 46%	38, 25%	13, 30%
	40–49	136, 29%	71, 27%	51, 33%	14, 32%
	50–59	67, 14%	27, 10%	39, 25%	1, 2%
	60 and older	13, 3%	5, 2%	2, 1%	6, 14%
3. Job title		464	267, 58%	153, 33%	44, 9%
4. Institution size	University hospital or medical center	281, 61%	174, 65%	81, 53%	26, 59%
	Regional general hospital (>300 beds)	147, 32%	72, 27%	61, 40%	14, 32%
	Other	36, 8%	21, 8%	11, 7%	4, 9%
5. Career experience, years	1–5	82, 18%	52, 19%	20, 13%	10, 23%
	6–10	79, 17%	58, 22%	16, 10%	5, 11%
	11–15	95, 20%	63, 24%	23, 15%	9, 20%
	16–20	71, 15%	36, 13%	27, 18%	8, 18%
	More than 21	137, 30%	58, 22%	67, 44%	12, 27%
6. Operation of the fluoroscopy unit	Yes	325, 70%	246, 92%	50, 33%*	29, 66%*
7. Use of lead apron	Yes	457, 98%	266, 100%	152, 99%	39, 89%^†^
8. Use of thyroid collar	Yes	123, 27%	71, 27%	42, 27%	10, 23%
9. Use of lead glass	Yes	164, 35%	103, 39%	49, 32%	12, 27%
10. Use of radiation dosimeter	Yes	344, 74%	160, 60%	144, 94%*	40, 91%*
11. Fluoroscopy unit type	I do not know	54, 12%	26, 10%	25, 16%	3, 7%
12. RE of each procedure	Yes	84, 18%	45, 17%	15, 10%	24, 55%*
13. Basic lecture on RE	Yes	351, 76%	215, 81%	98, 64%^‡^	38, 86%
14. Three principles of RP	Yes	338, 73%	209, 78%	96, 63%§	33, 75%
15. DRL	Yes	84, 18%	46, 17%	12, 8%**	26, 59%*
16. RE dose increasing cancer rate by 0.5%	100 mSv	292, 63%	180, 67%	88, 58%	24, 55%
17. Revised lens dose 5‐year limit	100 mSv	262, 56%	158, 59%	77, 50%	27, 61%

Abbreviations: DRL, diagnostic reference level; RE, radiation exposure; RP, radiation protection.

* *p* < 0.0001, ^†^ *p* = 0.0002, ^‡^ *p* = 0.0003, ^§^ *p* = 0.0009, ^**^ *p* = 0.0078, compared to doctors.

### Differences according to job title

Regarding background, nurses were significantly older (*p* = 0.0003), but the age trend of technicians was not different (*p* = 0.2731) from that of doctors. No significant differences in hospital type or experience trends were observed between doctors, nurses, and technicians.

**FIGURE 1:**
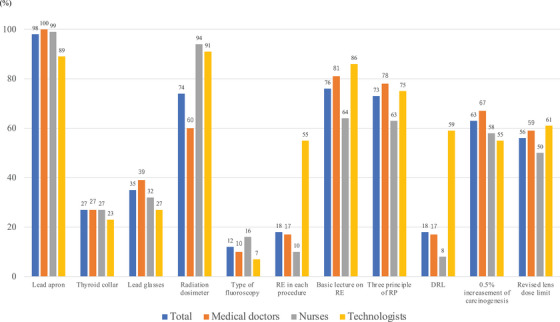
Differences by the profession.

Regarding equipment, the wearing lead apron rates for doctors, nurses, and technologists were 99.6%, 99.4%, and 88.6%, respectively. Compared to medical doctors, there was a significantly lower rate of wearing lead aprons technologists (*p* = 0.0002). The rates of thyroid collar use were equally low in all occupations, 27%, 27%, and 23% in doctors, nurses, and technologists, respectively. Similarly, the rates of lead glasses were low at 39%, 32%, and 27%, respectively. The rates of radiation dosimetry in doctors (60%) were significantly lower than those in nurses (94%, *p* < 0.0001) and technologists (91%, *p* < 0.0001). The rates of radiation dosimeters were significantly lower (60%) for doctors than for nurses (94%, *p* < 0.0001) and technicians (91%, *p* < 0.0001; Table 2 and Figure 1). Similar to the previous survey, medical doctors had a significantly lower rate of dosimeter use than the other medical workers (*p* < 0.0001). Regarding knowledge of radiation protection, 26 doctors (10%), 25 nurses (16%), and three technologists (7%) did not know the type of fluoroscopy unit in their workplaces. In total, 45 medical doctors (17%), 15 nurses (10%), and 24 technologists (55%) were aware of the radiation dose for each procedure. Significantly more technologists knew the dose (*p* < 0.0001). Two hundred fifteen medical doctors (81%), 98 nurses (64%), and 38 technologists (86%) had received lectures on radiation protection, but the number of nurses was significantly lower (*p* = 0.0002). In total, 209 medical doctors (78%), 96 nurses (63%), and 33 technologists (75%) were aware of the three principles of radiation protection, although the number of nurses was significantly lower (*p* = 0.0002).

## DISCUSSION

This survey was intended to follow up on the previous survey to determine the behavioral change in the interim. Previous and current surveys were conducted anonymously to encourage respondents to provide accurate information while protecting their privacy. Although some one‐off surveys of medical staff have been conducted, there have been no reports of changes over time. Strictly, since this survey was not the same cohort as the previous 2020 study, it is not statistically possible to compare them, but it is possible to consider the results as a general trend over the past 2 years because the hospitals surveyed are nearly identical to the previous cohort. Since there was a large enough sample size, it is possible to evaluate change over the past 2 years, which is a strength of our survey.

The latest survey revealed that the rate of lead apron use was sufficient (98%), but the rates of thyroid collar use (27%) and lead glasses use (35%) were low (Table 2). Figure S1 shows the improvement in the rates of wearing lead glasses, which was 39% in doctors and had increased by 18% (Figure S1). Regarding the use of lead glasses, improvements were seen in university hospitals or center hospitals (+22%) and other hospitals (+20%), but not enough improvement was observed in general hospitals (+1%) based on hospital size. Additionally, improvement was noticeable in those who were of a younger age and operated fluoroscopy units (Figure S2). Information dissemination through participation in the previous survey^9^ and the REX study^11^ may have led to improvement. The coronavirus disease 2019 outbreak may have posed challenges to acquiring the volume of eye‐face infection guards and protective equipment needed. Except for doctors, the rate of wearing protective equipment seemed to show no changes or deterioration. However, since the number of nurses and technicians is small compared to doctors and the cohort is not exactly the same as the previous cohort, it is difficult to determine whether compliance is better or worse.

Dosimeter wearing was extremely low among doctors (60%), unlike nurses (94%) and technologists (92%), which was similar to a previous report^9^ and showed improvement by 8% from 52% of previous surveys but still is not enough (Figure S1). Similarly, surveys in other countries have reported low rates of doctors wearing dosimeters, and we believe that this is a common trend among doctors.^6^, ^12^ In particular, this trend may only be observed by endoscopists.^13^, ^14^ It may depend on differences in the medical department due to differences in the educational environment and mandates of the major medical societies rather than the efforts of each individual.

Knowledge and education are essential for radiation protection, as the ICRP has long advocated.^2^ We found that 12% of participants did not know about the type of fluoroscopy, over or under the couch, in their institutions. Since the prediction of scattered rays differs between over and under the couch, it is necessary to know their characteristics to avoid unnecessary REX. Radiation exposure is particularly high between the irradiation and the target. In over couch, the use of a protective shield is effective.^15^, ^16^, ^17^, ^18^ However, in endoscopic procedures, there are many situations where staff manually control dangerous body movements to ensure patient safety because the patient is sedated. In such cases, all staff members need to be aware of the high risk of irradiation, especially, without a protective shield over the couch.

In this study, we found that the awareness of radiation protection seemed to improve. Between the two surveys, the 2019 Ordinance for Enforcement of the Medical Care Act was amended, effective April 1, 2020. This could have improved awareness about radiation protection. The breakdown showed that the basic lecture on REX and the three principles of radiation protection were approximately 80%, which was increased, especially among technologists, from 25% to 36% (Figure S1). However, the rates of awareness of REX for each procedure and DRL were lower for doctors (17% and 17%, respectively) and nurses (10% and 8%, respectively), but technicians were more aware (55% and 59%, respectively). The concept of DRL is to understand the exposure dose for a procedure and judge whether it is appropriate or not. Although most doctors operate fluoroscopy units, only 17% of them know the RE for each procedure. We found that it is necessary to further inform doctors and nurses of the DRL concept. There have been similar reports about the lack of knowledge.^7^, ^19^, ^20^ The awareness of 0.5% increased mortality per 100 mSv, and the revised lens dose 5‐year limit^21^ was approximately 50%–60%. In particular, the eye lens REX dose of the 5‐year limit represents 20 mSv per year, which is approximately one‐fifth of the previous level. Therefore, it is advisable to reconfirm with staff the current situation and RP principles such as protective goggles, shields, and distance.

In conclusion, there was some improvement, but not enough over a 2‐year period. Radiation protection was still insufficient, as in the previous survey, although we performed the REX study with almost the same population. It may be worth involving gastroenterology‐ or endoscopy‐related academic societies in informing the public and making safety protocols mandatory.

## CONFLICTS OF INTEREST STATEMENT

The author Mamoru Takenaka is an AE of Digestive Endoscopy. The author Ichiro Oda is an AE of Digestive Endoscopy. The author Yousuke Nakai is an AE of Digestive Endoscopy. The author Seiichiro Abe is an AE of DEN Open. The rest of the authors declare no conflict of interest.

## ETHICS STATEMENT

All the participants were informed of the study protocol. After providing informed consent by checking the agreement box in the web‐based questionnaire, the participants were enrolled in the study. This study was performed in accordance with the guidelines of the Declaration of Helsinki. Ethics approval was not sought for the present study because of the nature of the anonymous questionnaire survey.

## Supporting information



Figure S1

Figure S2
