# Correction to “A follow‐up questionnaire survey 2022 on radiation protection among 464 medical staff from 34 endoscopy–fluoroscopy departments in Japan’’

**DOI:** 10.1002/deo2.244

**Published:** 2023-05-25

Hayashi S, Takenaka M, Kogure H *et al.* A follow‐up questionnaire survey 2022 on radiation protection among 464 medical staff from 34 endoscopy–fluoroscopy departments in Japan. *DEN Open* 2023; **3**: e227.

There was an error in the seventh author's name. It should be “Yamaguchi S”.

The online article has been amended.

The authors apologize for this error.

