# Supplementary material for: A follow‐up questionnaire survey 2022 on radiation protection among 464 medical staff from 34 endoscopy–fluoroscopy departments in Japan
Source: DEN Open. 2023 Apr 13;3(1):e227. doi: 10.1002/deo2.227 (PMC10102737; doi:10.1002/deo2.227)
Supplement: Supplementary file 1 — Figure S1 [file DEO2-3-e227-s001.pdf]

Question [answer], difference (2022, 2020) (%)	All	Medical doctors	Nurses	Technologists
Lead apron [Yes]	-1 (98, 99)	0 (100, 100)	0 (98, 98)	-9 (89, 98)
Thyroid collar [Yes]	-5 (27, 32)	0 (27, 27)	-12 (27, 39)	-15 (23, 38)
Lead glasses [Yes]	+14 (35, 21)	+18 (39, 21)	+12 (32, 20)	+2 (27, 25)
Radiation dosimeter [Yes]	+5 (74, 69)	+8 (60, 52)	0 (94, 94)	-1 (91, 92)
Type of fluoroscopy unit [I do not know]	+1 (12, 11)	+3 (10, 7)	-3 (16, 19)	-1 (7, 8)
RE in each procedure [Yes]	+3 (18, 15)	+4 (17, 13)	-3 (10, 13)	+17 (55, 38)
Basic lecture on RE [Yes]	+12 (76, 64)	+10 (81, 71)	+10 (64, 54)	+36 (86, 50)
Three principles of RP [Yes]	+14 (73, 59)	+17 (78, 61)	+4 (63, 59)	+25 (75, 50)

Supplementary Figure 1
