# Supplementary material for: A follow‐up questionnaire survey 2022 on radiation protection among 464 medical staff from 34 endoscopy–fluoroscopy departments in Japan
Source: DEN Open. 2023 Apr 13;3(1):e227. doi: 10.1002/deo2.227 (PMC10102737; doi:10.1002/deo2.227)
Supplement: Supplementary file 2 — Figure S2 [file DEO2-3-e227-s002.pdf]

Lead glasses [Yes]

Institutions	University or Center hospital	General hospital (>300 beds)	Others		
difference (2022, 2020) (%)	+22 (37, 15)	+1 (33, 32)	+20 (31, 11)		
number/ total	105/281, 20/135	48/147, 53/110	11/36, 4/37		
**Age**	**20's**	**30's**	**40's**	**50's**	**Over 60**
difference (2022, 2020) (%)	+22 (38, 16)	+21 (38, 17)	+12 (37, 25)	+1 (24, 23)	-36 (31, 67)
number/ total	28/74, 7/45	66/174, 18/105	50/136, 26/103	16/67, 6/26	4/13, 2/3
**Fluoroscopy operation**	**Yes**	**No**			
difference (2022, 2020) (%)	+18 (38, 20)	+6 (28, 22)			
number/ total	125/325, 38/188	39/139, 21/94			

Supplementary Figure 2
